# The influence of parenting style on academic achievement and career path

**Published:** 2016-07

**Authors:** ZAHRA ZAHED ZAHEDANI, RITA REZAEE, ZAHRA YAZDANI, SINA BAGHERI, PARISA NABEIEI

**Affiliations:** 1 Khorasgan (Isfahan) Branch, Islamic Azad University, Isfahan, Iran;; 2 Education Development Center, Quality Improvement in Clinical Education Research Center, Shiraz University of Medical Sciences, Shiraz, Iran;; 3 Department of Educational Management, College of Education Science, Khorasgan (Isfahan) Branch, Islamic Azad University, Isfahan, Iran; 4 Student Research Committee, Shiraz University of Medical Sciences, Shiraz, Iran

**Keywords:** Parenting, Achievement, Career, Students

## Abstract

**Introduction:**

Several factors affect the academic performance of college students and parenting style is one significant factor. The current study has been done with the purpose of investigating the relationship between parenting styles, academic achievement and career path of students at Shiraz University of Medical Sciences.

**Methods:**

This is a correlation study carried out at Shiraz University of Medical Sciences. Among 1600 students, 310 students were selected randomly as the sample. Baumrind’s Parenting Style and Moqimi’s Career Path questionnaires were used and the obtained scores were correlated with the students' transcripts. To study the relation between variables Pearson correlation coefficient was used.

**Results:**

There was a significant relationship between authoritarian parenting style and educational success (p=0.03). Also findings showed a significant relationship between firm parenting style and Career Path of the students, authoritarian parenting style and Career Path of the students, educational success and Career Path of the students (p=0.001).

**Conclusion:**

Parents have an important role in identifying children’s talent and guiding them. Mutual understanding and close relationship between parents and children are recommended. Therefore, it is recommended that the methods of correct interaction of parents and children be more valued and parents familiarize their children with roles of businesses in society and the need for employment in legitimate businesses and this important affair should be more emphasized through mass media and family training classes.

## Introduction

Family is the fundamental and important structure of the society that has an important role in one's life and in the society. The importance of the family as a social structure is something unmistakable. Although affected by society and peers, children are more influenced by the family. The influence of the family on the child and its roles in the creativity, cultural, social, and moral aspects are very great and important. Correct and balanced relationship between parents and their children is one of the factors influencing both their physical and mental health. Research has shown that interaction between children and parents and how parents communicate with children are considered to be the most important and fundamental factors among the various factors that affect children’s fostering and healthy character (1).

The relationship of parents with children or parenting style serves multiple purposes. Moral and psychological training, identification, growth and development of children's talents, skills, familiarizing with the rules and norms of the society from the perspective of parents are among these purposes. "Parsons also consider two basic functions for the family, i.e. socialization and prosperity of the child's personality." So it seems that parents’ parenting styles are likely to affect children's personality traits (2).

Parenting styles can be defined as a set or a system of behaviors that describes the parent and child interactions over a wide range of situations and creates an effective interaction atmosphere (3). Parenting style is a determining and effective factor that plays an important role in children’s psychopathology and growth (4). In the present study, ponderable points in parenting styles, physical presence of parents at home, i.e. the time to be with the children and cultural spaces of the family are considered.

Educational achievement means the fulfillment of expected level of education, and an education organization approaches its predetermined goals. Educational achievement means increase of learning, increase of the level of good scores and admission of students in the courses and educational grades (5, 6).

Traditionally, career success is defined for those who receive good salaries for their jobs or have high positions and positions with more favorable responsibilities, motivation, adequate distinction, and progress. The Employees and managers are expected alike to have a commitment to the organization. It is assumed that if the employees have appropriate jobs which they are loyal to, the management offers them the rewards such as promotion, fringe benefits, job security and more respect and even work authority.  Thus, the labor force of the organization will feel dignity (7, 8).

Offering more job and education information and helping each student with self-image during the education can give him the logical principles to make a correct decision. Many people make decisions about their careers by observing the world around and try to match themselves with it. This is something contrary to what should be really done. The best career path is a process that begins within (a kind of evolution of self-consciousness). Individuals have to be aware of their skills, talents, abilities, capabilities and unique features because this self-consciousness is a cycle which leads the individual towards a satisfactory job. Choosing a profession proportionate to individual’s interests and abilities is one of the most important stages of life for all people (7).

In fact, all of us, not only earn money from our chosen career, but also determine our social status. Therefore, we can expect that, by choosing our profession, we determine our social status and self-respect in addition to the income level. As noted, people entering the world of work and organizations are seeking growth and progress, thus, they choose the path that has been designed by the manager’s discretion. The task of management is to determine career progress path, establish compatibility between needs, potential abilities of the people on the one hand and, on the other hand, professional needs of the organization and determine career progress path of each individual during his career. Management of career path development is one of the activities of human resources that lead each individual to ideal perfection through finding his/her progress path in the working life. This fact leads to job satisfaction and professional tenure and higher effectiveness (7).

But, the question that comes to mind is that whether the educational success relates to parents’ parenting styles. And secondly, what is the relationship of parenting styles with students' career path? 

No quite similar studies on the subject of the present study have been done so far. Therefore, this study seeks to examine the relationship between parents’ parenting styles and educational success and career path of the students of Shiraz University of Medical Sciences in the academic year 2014. In this study, we intended to find out whether there is a relationship between parenting styles and students’ academic achievement and career path? The results of the previous researches suggest this relationship.

According to Sanaee (2008) career choice is one of the most important events in life that affects every aspect of human existence. In the theories of career choice, factors affecting career choice are studied and help the individual to choose an appropriate job which leads to personal satisfaction and increase of the efficiency. The results of the study showed that there was a significant relationship between self-concept and job satisfaction, as well as career self-concept and job satisfaction (9).

Mehrafza (2005) in a study examined the relationship between parenting styles and creativity and academic achievement of the students of grade three of high school and showed that there was a significantly positive relationship between the emotional atmosphere of the family, declining to the principles of democracy, and creativity. Furthermore, there was a significantly negative relationship between the creativity and dictatorship principles and there was no statistically significant relationship between the emotional atmosphere of the family, declining to absolute freedom, and creativity (10).

Abedi et al. (2005) in a study examined the relationship between motivations of educational achievement of high school students of Isfahan and their family characteristics. The results showed that from among family factors associated with educational achievement, motivation, parents’ expectations of children's success (0.28), authoritarian parenting style (0.26) and family structure (modern natural family) (0.16) explain educational achievement motivation (11).

Biabangard (2005) in a study examined the relationship of self-esteem and motivation with educational achievement among students of grade three of high school in Tehran. The researcher found that there was a significant correlation between self-esteem, achievement motivation, and educational achievement, between self-esteem and achievement motivation, between self-esteem and educational achievement, and between achievement motivation and educational achievement. There was no significant difference between self-esteem, achievement motivation, and educational achievement of both groups of male and female students of the fields of the Humanities and Experimental Sciences (12).

Kefayat (1994) conducted a study titled "Examination of the relationship of parenting styles and attitudes with creativity and its relationship with intelligence, educational achievement and progressivist behaviors of students of the first grade of high school in Ahvaz" and concluded that there was a negative correlation between the various parenting styles and creativity (13).

## Methods

This is a correlation study which is done at Shiraz University of Medical Sciences. Among 1600 students, 310 students were selected randomly as the sample. Baumrind’s Parenting Style and Moqimi’s Career Path questionnaires were used and also to explain the relationship between the three variables, students' transcripts were used to determine the level of educational success. Regarding the study population, stratified random sampling method was used. Library, the Internet, local and foreign papers and studying the theoretical principles of the subject were used for access to the records. In this study, the following questionnaires were used:

- Baumrind parenting style questionnaire
- Moqimi career path questionnaire

The GPAs of senior students of various fields of study in Shiraz University of Medical Sciences in the academic year 2014 were used to determine the educational success. 

To determine the face and content validities of the questionnaires, the primary questionnaires were given to 3 professors and experts and after getting their opinions, the necessary changes were applied on the questionnaire, thus, the items of the questionnaires were modified and confirmed considering professors’ opinions.  A test is reliable if it is given to the same group of people for several times in a short time, and the results are consistent. After analyzing the data from the questionnaires, their reliability coefficient (Cronbach's Alpha) was approved. Regarding the Parenting Styles Questionnaire: for permissive style, authoritarian style, and firm style, the reliability coefficients were respectively 0.72, 0.80, and 0.85 and for the Career Path Questionnaire: α = 0.82. 

## Results

310 students participated in the study, 194 were male and 116 were female. In fact, the majority of subjects were male students, i.e. 62.6%, and female 37.4%. The majority of subjects were 21 to 25 years old (46.1%), the students under 20 years: (29%), those from 26 to 30 years: 18.1% and finally students over 30 years of age: 6.8%. More than half of the subjects were studying at B.S. level (61%), 21.3% at A.S. level, and 17.7% at M.S. level or higher. 

The correlation matrix between the studied variables is presented in the following table that shows the correlation coefficients between all variables (Table 1). According to the table, there is a positive and significant relationship between firm and reassuring parenting style and educational success and career path at the significance level of 0.01, while the authoritarian parenting style has a negative and significant relationship with educational success and career path at 0.05. There is no significant relationship between permissive parenting style and these two variables.

**Table 1:** Correlation coefficients between parenting styles and educational success and career path of the students

**Variable**	**Firm style**	**Authoritarian style **	**Permissive style**	**Educational success**	**Career path**
**Firm style**	1				
**Authoritarian style**	-0.20*	1			
**Permissive style**	0.02	-0.56**	1		
**Educational success**	0.62**	-0.16*	-0.05	1	
**Career path**	0.68**	-0.26*	-0.10	0.47**	1

**significant at p<0.01

*significant at p<0.05

The correlation coefficient between firm and reassuring parenting styles and educational success is 0.62 with the significance level of 0.001, which is less than 0.05. In other words, this result shows that there is a significant relationship between the parents’ firm parenting style and the students’ educational success and this relationship is positive and direct. The correlation coefficient obtained shows that the relationship between these two variables is relatively high. 

The results of correlation between authoritarian parenting style and educational success show that there is a significantly negative relationship between the parents’ authoritarian parenting style and the students’ educational success (p=0.03). 

The results of the examination of the relationship between permissive parenting style and students’ educational success, show that this relationship is not statistically significant (p=0.36).

The results of correlation between firm parenting and career path show that there is a significantly positive relationship between parents’ firm parenting style and students’ career path. This means that when the score of the firm and reassuring parenting style increases, the score of career path increases, too, and vice versa.

Other results show that there is a significantly negative relationship between parents’ authoritarian parenting style and students’ career path. This means that the more the score of authoritarian parenting styles of parents, the less the students’ score of career path will be, and vice versa. 

The result of the examination of the relationship between permissive parenting styles and students’ career paths shows that this relationship is not statistically significant since the significance level is 0.08 which is greater than 0.05. 

The correlation coefficient between educational success and career path is 0.47 and the significance level is 0.001; therefore, the research hypothesis is confirmed and we can say that the relationship between educational success and students’ career path is statistically significant. The correlation coefficient, i.e. 0.47, also suggests moderate relationship. 

## Discussion

The results show that autonomy, parental involvement and warmth are significant predictors for academic achievement. Also there is a positive significant relationship between firm parenting style and student’s academic achievement. 

Other results indicate that the high success in education is strongly associated with parenting styles. It has been found that very successful students at school with high scores had parents with firm styles. Those students with authoritative parents had the least scores and it has an acceptable conformity with the results of this study. The findings of this study also have conformity with Mehrafza’s (10, 14).

Mehrafza (2005) in a study examined the relationship between parenting styles and creativity and educational achievement of the students of grade three of high school and found that there was a significantly positive relationship between the emotional atmosphere of the family, declining to the principles of democracy, and creativity. Furthermore, there was a significantly negative relationship between the creativity and dictatorship principles and there was no statistically significant relationship between the emotional atmosphere of the family, declining to absolute freedom, and creativity which is in an acceptable conformity with the results of this study (10).

The results showed that there was a significantly positive relationship between parents’ firm parenting style and students’ career path. This means that when the score of the firm and reassuring parenting style increases, the score of career path increases, too, and vice versa. The correlation coefficients obtained shows that the relationship between these two variables is relatively high. 

Other studies have also investigated the relationship between the economic status of students' families and their educational tendencies so that the students with better economic status were more willing to have university education. Also, the children of more educated families were more willing to have university education and related occupations. In addition, family members have a more determining role in all of student’s educational-occupational aspects than the other groups. Other than families, students consult school authorities and their friends for the educational and occupational affairs (15-17).

## Conclusion

Children’s choice of a future career depends on many factors including the parenting styles and their education. If the child's mind is active during his development about his future career through career counseling programs, in the secondary school the child gains the necessary knowledge and information about himself, jobs and his cognitive world is organized about businesses and in high school, he learns the job search process, along with the skills thereof and has a practical relevance with the world of businesses and also continues this process at the university and also begins useful work experience at this time. So selecting a successful career proportionate to the needs and talent and personality of the person will not be inaccessible. 

Parents have an important role in identifying children’s talent and guiding them. Mutual understanding and close relationship between parents and children are recommended. Therefore, it is recommended that the methods of correct interaction of parents and children be more valued and parents familiarize their children with roles of businesses in society and the need for employment in legitimate businesses and this important affair should be more emphasized through mass media and family training classes. 

**Recommendations**

- Since the research sample is limited to the students, it is likely that factors such as age, education, culture, social situation, workplace, etc., influence educational achievement; therefore, it is recommended to consider these factors in the same future studies. 
- Present direct and indirect training of parenting styles for parents to support students’ progress and responsibility and consequently career path.
